# AI-Radiomics Can Improve Inclusion Criteria and Clinical Trial Performance

**DOI:** 10.3390/tomography8010028

**Published:** 2022-02-02

**Authors:** Michal R. Tomaszewski, Shuxuan Fan, Alberto Garcia, Jin Qi, Youngchul Kim, Robert A. Gatenby, Matthew B. Schabath, William D. Tap, Denise K. Reinke, Rikesh J. Makanji, Damon R. Reed, Robert J. Gillies

**Affiliations:** 1 Department of Cancer Physiology, H. Lee Moffitt Cancer Center and Research Institute, Tampa, FL 33612, USA; em.tomaszewski@gmail.com (M.R.T.); jean_fsx@hotmail.com (S.F.); algarci2@mail.usf.edu (A.G.); Jin.qi@moffitt.org (J.Q.); 2 Department of Radiology, Tianjin Medical University, Tianjin 301700, China; 3 Department of Biostatistics, H. Lee Moffitt Cancer Center and Research Institute, Tampa, FL 33612, USA; Youngchul.kim@moffitt.org; 4 Department of Radiology, H. Lee Moffitt Cancer Center and Research Institute, Tampa, FL 33612, USA; Robert.Gatenby@moffitt.org; 5 Department of Cancer Epidemiology, H. Lee Moffitt Cancer Center and Research Institute, Tampa, FL 33612, USA; matthew.schabath@moffitt.org; 6 Department of Medicine, Memorial Sloan Kettering Cancer Center, New York, NY 10065, USA; tapw@mskcc.org; 7 Department of Medicine, Weil Cornell Medical College, New York, NY 10021, USA; 8 Sarcoma Alliance for Research through Collaboration, Ann Arbor, MI 48106, USA; dreinke@sarctrials.org; 9 Sarcoma Department, H. Lee Moffitt Cancer Center and Research Institute, Tampa, FL 33612, USA

**Keywords:** sarcoma, radiomics, enrichment strategy, trial design, doxorubicin, evofosfamide

## Abstract

*Purpose:* Success of clinical trials increasingly relies on effective selection of the target patient populations. We hypothesize that computational analysis of pre-accrual imaging data can be used for patient enrichment to better identify patients who can potentially benefit from investigational agents. *Methods:* This was tested retrospectively in soft-tissue sarcoma (STS) patients accrued into a randomized clinical trial (SARC021) that evaluated the efficacy of evofosfamide (Evo), a hypoxia activated prodrug, in combination with doxorubicin (Dox). Notably, SARC021 failed to meet its overall survival (OS) objective. We tested whether a radiomic biomarker-driven inclusion/exclusion criterion could have been used to improve the difference between the two arms (Evo + Dox vs. Dox) of the study. 164 radiomics features were extracted from 296 SARC021 patients with lung metastases, divided into training and test sets. *Results:* A single radiomics feature, Short Run Emphasis (SRE), was representative of a group of correlated features that were the most informative. The SRE feature value was combined into a model along with histological classification and smoking history. This model as able to identify an enriched subset (52%) of patients who had a significantly longer OS in Evo + Dox vs. Dox groups [*p* = 0.036, Hazard Ratio (HR) = 0.64 (0.42–0.97)]. Applying the same model and threshold value in an independent test set confirmed the significant survival difference [*p* = 0.016, HR = 0.42 (0.20–0.85)]. Notably, this model was best at identifying exclusion criteria for patients most likely to benefit from doxorubicin alone. *Conclusions:* The study presents a first of its kind clinical-radiomic approach for patient enrichment in clinical trials. We show that, had an appropriate model been used for selective patient inclusion, SARC021 trial could have met its primary survival objective for patients with metastatic STS.

## 1. Introduction

In the last decade, there has been an explosion in the use of advanced image analysis with machine learning, known as “Radiomics” [1,2]. Radiomic analyses of cancer can be used to stage, prognose patient outcome, predict response to specific therapies and, most recently, to inform therapeutic choices [3] with increasing connectivity between image features and tumor biology [4]. However, this promising method has to date not been able to compare two treatments and choose an optimal therapeutic approach or identify patients likely to benefit from one drug over another. We aimed to develop an appropriate model, allowing for radiomic approaches to be used in clinical trials for patient enrichment. We tested this hypothesis in a retrospective analysis of data from the SARC021 [5] phase III clinical trial in metastatic soft tissue sarcoma that compared overall survival (OS) in cohorts treated with doxorubicin (Dox) to those treated with Dox + Evofosfamide (Evo), a hypoxia activated pro-drug of a brominated version of isophosphoramide mustard (NCT01440088). Although Dox + Evo had shown promise for sarcoma in phase II [6], the phase III trial failed to meet its threshold of increased OS in the Dox + Evo cohort [5]. 

Soft tissue sarcomas are a heterogeneous group of malignancies originating in mesenchymal tissue that commonly metastasize to the lungs, with an associated poor prognosis [7]. An historical median OS of 12 months for metastatic soft tissue sarcoma patients has steadily improved to 20.4 months on trials, which may be attributed to better patient selection along with better supportive care and additional options in second line and therapies beyond [8,9,10]. In the SARC021 trial, the shifting survival with Dox monotherapy led to the study being under powered [11]. *Biomarkers that can exclude patients who are likely to benefit from standard therapy would thus be useful to focus trials on those most likely to benefit from an experimental therapeutic approach.* In this first of its kind study, we present a novel analytic framework that can identify patients most and least likely to benefit from trial enrollment. Radiomic feature extraction is combined with customized statistical analysis to create a risk score. This score as iteratively analyzed to identify a threshold value to identify subjects predicted to benefit from Dox monotherapy. These represented 48% of the patients on the trial and, if they were excluded from enrolling, there was a significant (*p* < 0.05) difference in OS between three Dox and Dox + Evo arms of the study. It is conceivable that such biomarker could be used to exclude subjects in other STS interventional trials.

## 2. Materials and Methods

### 2.1. Patient Populations

This study was approved by the University of South Florida Institutional Review Board. The analysis includes patients who participated in the TH CR-406/SARC021 multicenter clinical trial of Doxorubicin plus Evofosfamide (Dox + Evo) versus Doxorubicin alone (Dox) in locally advanced, unresectable or metastatic soft-tissue sarcoma. Full trial protocol and results were published by Tap et al. [5]. A total of 640 patients were enrolled. The primary endpoint of the trial was OS. Survival and clinical data were available for 607 patients, and CT images obtained prior to treatment were available for analysis in 581 patients.

### 2.2. Patient Data and CT Images

Patient covariates and CT image were obtained from the Sarcoma Alliance for Research through Collaboration (SARC). The CT images were uploaded into HealthMyne Quantitative Imaging Decision Support (QIDS) software (QIDS, Madison, WI, USA), where a radiologist with 10 years of experience (S.F.) identified and segmented all visible lesions. 346 patients were found to have at least one lesion in the lung, the most common metastatic site in the considered cohort (followed by liver lesions, identified in 106 patients), as anticipated [7]. Only lung patients were included in the study to enable comparison of image features between individuals, and hence the use of radiomics. Of these patients, 296 had contrast enhanced CT scans of the lung which could be analyzed, and these were used for quantification. This total cohort of 296 patients used in this study was randomly divided 70:30 into training and test sets using the “sample” function in R version 4.0.2. The test set was sequestered until the final model was developed in the training set for its most stringent validation and increased reproducibility compared to cross-validation approaches [12]. Robustness of the feature selection to the training/test split was confirmed as described in Supplementary Materials.

### 2.3. Radiomic Feature Extraction

Anonymized imaging data and segmentation structures in DICOM format were retrieved from Healthmyne servers. Details of image pre-processing are described in Supplementary Materials. For each patient, a total of 163 features were calculated in 3D using standardized algorithms from the Image Biomarker Standardization Initiative (IBSI) v5 [13]. The radiomic features included statistical, histogram, shape & size, Grey Level Cooccurrence Matrix (GLCM), Grey Level Run Length Matrix (GLRLM), Grey Level Size Zone Matrix (GLSZM) and Neighboring Grey Tone Difference Matrix (NGTDM) features, as well as 16 peritumoral features as described before [14]. Laws and Wavelet features were not extracted due to their poor reproducibility reported in previous studies [15]. As standard in radiomic studies [16], to ensure the radiomic signatures provide additional information compared with tumor volume, the features strongly correlated to volume (Pearson |r| > 0.8) were excluded from further analysis, while volume itself was included. Spatial stability of the features was assessed, as described in the Supplementary Materials, and unstable features excluded. 

### 2.4. Feature Selection

The goal of this analysis was to identify the radiomic features and patient covariates differentially associated with OS in the two treatment groups, which was the primary endpoint in the original trial [5]. A new statistical framework was therefore developed.

First, univariable Cox proportional hazards regression analysis was used to assess the degree and direction of statistical association of each feature and covariate with post-treatment OS, separately in Dox and Dox + Evo arms. False discovery rate Benjamini-Hochberg [17] correction was applied to the *p* values of radiomic features to account for multiple testing. For each arm, features and covariates were considered promising in either of the two scenarios: (i) They showed significant association (*p* < 0.05) with survival in one treatment arm AND no association (*p* > 0.30) in the other arm, or (ii) they showed potential association (*p* < 0.30) with survival in both groups in opposite directions (HR > 1 in one group and <1 in the other). 

Correlations between the remaining features were calculated (Pearson’s correlation coefficient for continuous and Chi Square independence test statistics for categorical variables). For significantly correlated (*p* < 0.05) feature groups, features with lowest univariable Cox regression *p* value in the corresponding treatment group was retained as a representative of the group, and others excluded to avoid redundant information. If these *p*-values were exactly equal for several features due to the multiplicity correction, the *p*-values prior to multiplicity correction were compared. Of the remaining features and covariates, the one with lowest *p*-value ratio in the two treatment groups (low divided by high) was used in model training. 

### 2.5. Final Model Construction

The two final sets of features and covariates predicted to be most informative of the differential response to Dox or Dox + Evo were used to build the corresponding separate multivariable Cox proportional hazards regression models. Risk scores that are log-transformed relative risks of death were calculated using the “predict.coxph” function in R for all patients in the model training cohort and used to determine threshold for patient virtual inclusion and exclusion from the trial. The process of determining the optimum risk score threshold is described in the results section. Risk score values were predicted for all patients in the test cohort from the final multivariable Cox models constructed as above. The threshold values found to result in best separation of the treatment arms as found in the training set were applied to enrich the test cohort, and survival was compared between the treatment arms in the included subset of the test cohort using log-rank test. 

For the Dox model, where patients with high risk score values were expected to perform poorly under Dox, and thus be more likely to favor Dox + Evo treatment, a search for the optimal threshold was performed iteratively including sub-cohorts of patients with risk score above 1st, 2nd, 3rd etc. to 97th percentile of the total training cohort, evaluating survival difference between the treatment arms in terms of Cox regression *p*-value and hazard ratio each time. Thus, the entire range of possible thresholds was interrogated, to check if such selection can lead to significant treatment arm separation and identify an optimal threshold value.

## 3. Results

### 3.1. Patients

Clinical covariates included in the analysis are listed in Table 1, with their description included in Supplementary Table S1. Presence of lung metastasis was associated with significantly poorer overall survival in the entire cohort of 607 patients (*p* = 0.007, HR = 1.34 (1.09–1.65)). This was not the case for patients with liver metastases, which was the second most common metastatic site (*p* = 0.44). Among patients with lung metastases, no significant survival difference was observed between the two treatment groups (*p* = 0.8), similarly to the entire cohort (*p* = 0.45). Notably, the number of lung metastases in these patients was also not significantly associated with survival (*p* = 0.15).

No significant difference in OS was observed between the full training and test cohorts (*p* = 0.38, median OS: 17.0 (15.0–20.1) vs. 19.6 (14.0–26.9) training vs. test). No significant differences were also seen between Dox and Dox + Evo treatment groups in the training (*p* = 0.77, HR = 1.05 (0.76–1.46) median OS: 16.4 (12.1–20.6) vs. 17.1 (15.2–22.1) months Dox vs. Dox + Evo) or test cohorts (*p* = 0.37, HR = 0.80 (0.48–1.32) median OS: 23.3 (15.6–31.8) vs. 14.9 (11.1–27.2) months Dox + Evo vs. Dox).

### 3.2. Feature Stability

Concordance coefficients describing the spatial stability of the features were calculated, showing significant heterogeneity between and within feature classes. All results are visualized in Supplementary Figure S1 and detailed in Supplementary Table S2. As expected, shape features remained relatively unchanged, while statistical and histogram features were on average quite strongly affected by choice of ROI. Certain texture features, especially these related to Inverse Difference and Run Length, showed high robustness. Based on this exercise, 54 features with particularly poor robustness (CCC < 0.5) were excluded from further analysis. In addition, 12 features strongly correlated with tumor volume (Pearson Correlation Coefficient > 0.8) were represented by a single volume feature, leaving 81 intratumoral and 16 peritumoral features. 

### 3.3. Feature Selection

Univariable Cox proportional hazards regression was performed separately in the Dox and Dox + Evo treatment groups to identify radiomic features and clinical covariates differently associated with OS, are shown in Supplementary Table S3. Among clinical covariates, the histological classification of the primary tumor, tumor grade, and prior radiotherapy were significantly associated with longer survival and smoking history was significantly associated with shorter survival in the Dox cohort. None of these were significant in the Dox + Evo groups. Following elimination of correlated features, the clinical model retained histology (*p* = 0.010) and smoking history (*p* = 0.04).

No features or covariates were found to be significant in the Dox + Evo and not in the Dox group. Three uncorrelated radiomic features were found significantly associated with survival in the Dox but not in the Dox + Evo group: Short Run Emphasis, Normalized Run Length Nonuniformity and Small Zone Emphasis. Of these features, Short Run Emphasis, a measure of heterogeneity, showed the lowest ratio of *p* value in the Dox to the *p* value in Dox + Evo groups (*p* = 0.006 and *p* = 0.88 respectively), and was chosen for training a prediction model of post-treatment survival. 

### 3.4. Multivariable Model

**Figure 1:**
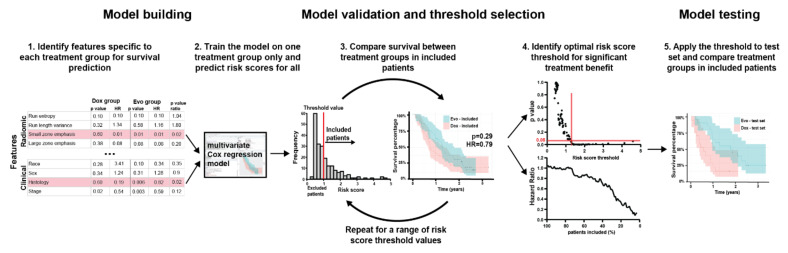
Patient inclusion model. Patient selection into the trial based on Dox group survival was executed according to the following method: firstly (**1**) radiomic and clinical features associated in training cohort with survival in Dox but not Dox + Evo treatment group were included in a multivariable Cox regression model (**2**), trained on Dox treated patients. The risk score assigned by the model to each training set patient was then used as a biomarker for inclusion into analysis, iteratively calculating the *p*-value and hazard ratio for survival comparison between treatment arms depending on minimum risk score threshold (**3**). If available, threshold corresponding to significant (*p*-value < 0.05) treatment benefit of Dox + Evo at highest percentage of patients included was chosen (**4**), and subsequently tested in the test cohort (**5**), with risk scores assigned by the multivariable Cox model developed in step (**2**). A corresponding model can also be developed based on Dox + Evo group survival, using a maximum risk score threshold.

**Figure 2:**
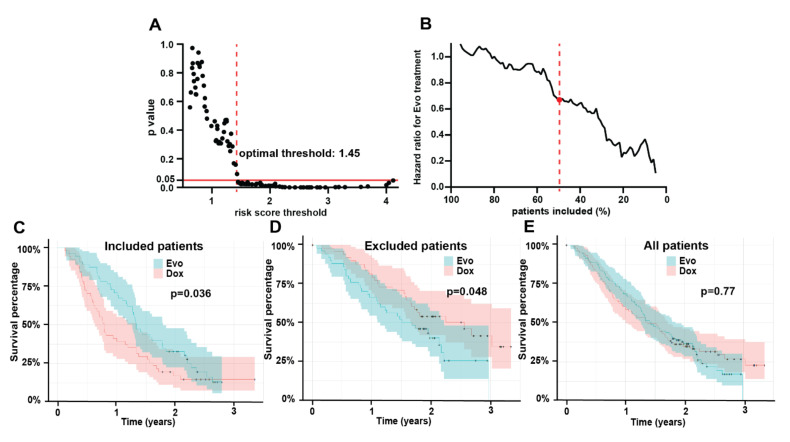
Multivariable Cox model enables selection of patients who benefit from Evofosfamide + Doxorubicin in training cohort. Quantification of the *p* value of overall survival difference in the training cohort between the Evofosfamide + Doxorubicin (Dox + Evo) vs. Doxorubicin alone (Dox) treatment arms depending on the minimum risk score for patient inclusion, as predicted by the model (**A**), shows a risk score threshold of 1.00 at which Doxorubicin + Evofosfamide (Dox + Evo) group shows significantly longer OS (*p* < 0.05). Exclusion of patients with high risk scores leads to monotonic decrease in the hazard ratio (**B**), and the 1.00 risk score threshold corresponds to inclusion of 52% of patients in the trial (indicated by red dotted line). The Kaplan-Meier plots by treatment arms show significantly better OS in the included (**C**) and significantly worse OS in the (**D**) excluded patients for the Dox + Evo treatment compared to Dox only. In all training set patients (**E**) no difference between the arms was observed.

The three features identified above (histology, non-smoking history, and radiomic Short Run Emphasis) were combined in a multivariable Cox model that was trained on the Dox cohort and produced a highly significant (*p* < 0.0001) signature of survival. Details of the model are shown in Supplementary Table S4. No significant correlation between residuals and time was measured (*p* = 0.46, 0.45, 0.71, 0.58 for SRE, histology, smoking history, and global test respectively) validating the proportional hazards assumption for Cox model use. No corresponding model was developed in the Dox + Evo group, as no clinical or radiomic features specific to this treatment arm were identified. The Dox model was used to predict risk scores for the entire training set, providing a predicted measure of risk of death if the Dox treatment was applied to all patients. *Patients with highest risk scores for Dox monotherapy (i.e., worse outcome) are expected to benefit the most from the alternative (Dox + Evo) treatment, and hence they should be included in the trial. Conversely, patients with a low Dox risk score should be excluded and undergo Dox monotherapy instead*. Such a patient enrichment strategy for the trial would thus be expected to result in an improved treatment benefit of Dox + Evo in the included patients. To assess this, we performed the log-rank test for difference in survival between Dox vs. Dox + Evo as a function of the risk score threshold for the remaining patients whose score was above that threshold. The schematic of the process is shown in Figure 1. As described in Supplementary Materials, the threshold separating high- from low-risk groups was incrementally increased to identify an optimum that reached a significant (*p* < 0.05) difference in OS, while including the largest fraction of patients. The results of this analysis are shown in Figure 2, demonstrating that increasingly different OS can be observed for the two treatment groups when patients with low-risk scores are excluded from virtual accrual (Figure 2A). The smaller *p*-values encountered with increasing thresholds were consistent with decreasing HR (Figure 2B), showing increasingly significant treatment benefit of Dox + Evo vs. Dox with more stringent inclusion criteria. A threshold of 1.45 allowed inclusion of 52% of the initial training cohort in the trial and showed a significant advantage of Dox + Evo over Dox [*p* = 0.036, HR = 0.64 (0.42–0.97)]. This result was visualized in divergent Kaplan Meier curves for the treatment groups in the included patients and longer survival in the Dox + Evo group (Figure 2C, median survival 16.0 (15.2–21.5) vs. 9.4 (7.6–16.0) months Dox + Evo vs. Dox) and a reverse trend for the excluded patients (Figure 2D, median survival 20.9 (13.2–26.6) vs. 30.7 (20.0-N.E) Dox + Evo vs. Dox, *p* = 0.036), with Dox treated group showing significantly longer survival, possibly related to the lack of Evo response in the subgroup and its added toxicity [5]. No difference was observed in the whole training cohort (Figure 2E). These figures make apparent that the most significant difference (*p* < 10^−4^) between included and excluded groups is their response to Dox monotherapy. Indeed, the difference in survival between included and excluded groups in the Dox + Evo trial arms was insignificant (*p* = 0.49).

A model using only clinical features as input was also trained and its performance evaluated as above. At the same inclusion rate as the radiomic-clinical model (52%), this approach does not show a significant survival difference between treatment arms (*p* = 0.20, HR = 0.78 (0.52–1.15)), reaching significance at a slightly lower proportion of included patients (48%, *p* = 0.02, HR = 0.59 (0.38–0.92) (see Supplementary Figure S2 for performance details). Although the Short Run Emphasis feature by itself did not significantly discriminate the groups (Supplementary Figure S3), it added to the significance of clinical features and thus increased the number of potential patients on trial from 48% to 52%. Selection based on lesion volume, routinely used in radiological analysis, could not separate patients likely to respond significantly differently to Dox and Dox + Evo treatments, neither through application of upper nor lower volume threshold (see Supplementary Figure S4).

### 3.5. Model Testing

**Figure 3:**
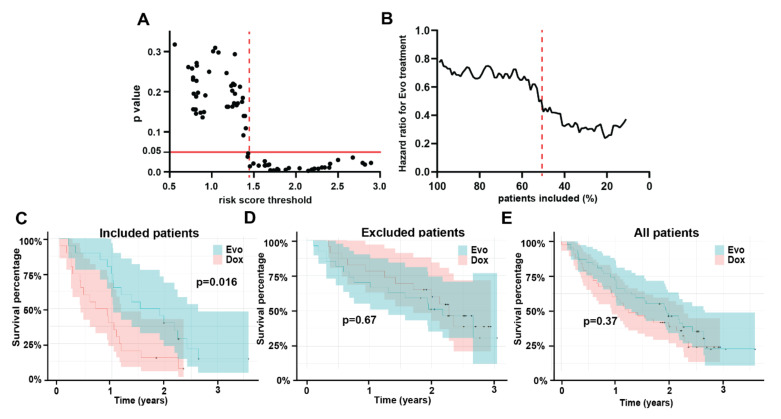
Results in the test cohort confirm the validity of the model. Risk scores predictions in the test cohort based on a multivariable Cox model trained on Dox treated training cohort patients can be used to identify patients who would benefit from Dox + Evo treatment. Graph in (**A**) shows that increasing the minimum risk score of patients included in the analysis leads to a stronger difference in survival between the treatment groups, as described by the *p* value of the comparison. For the risk score threshold of 1.45, a highly significant difference is observed (red point and dotted line), which corresponds to a decreased hazard ratio of the combination vs. standard therapy (**B**). These differences are apparent from the Kaplan-Meier curve in the included patients (**C**) showing significantly longer survival in the Dox + Evo group, while the excluded patients (**D**), or all test set patients (**E**) show no difference in survival between treatment groups.

The multivariable Cox model trained in the above section was used to predict risk scores for all patients in the test cohort. Similar to the training cohort, an increase in minimum risk score threshold for inclusion led to a monotonic decrease in *p* value (Figure 3A) and HR (Figure 3B) for the overall survival comparison between treatment groups. Applying the threshold of 1.45 determined *a priori* in the training set as the optimum threshold for inclusion, showed a significantly better survival in the Dox + Evo vs. the Dox treated group [*p* = 0.016, HR= 0.42 (0.20–0.85) Figure 3C]. As in the training cohort, this was significantly associated with an increased median survival of 20.6 months (12.3–31.5) for Dox + Evo vs. 9.6 (4.9–14.0) for Dox. As shown in Figure 3D the differences in the two treatment arms for the remaining excluded patients was insignificant for both OS (*p* = 0.67) and median survival 26.0 (15.6–N.E) vs. 27.2 (20.4–N.E), similar to the starting whole test cohort (Figure 3E). In the test cohort, applying the threshold of 1.44 resulted in inclusion of 50% of the subjects. As in the training set (Figure 2C,D), the selection by risk score threshold separated patients who did and did not respond to Dox (*p* < 10^−3^), whereas it did not discriminate (*p* = 0.27) responses of the Dox + Evo group. The plot of *p*-value vs. inclusion threshold (Figure 3A) shows a matching profile of improving treatment benefit of the Dox + Evo treatment (because of decreasing effectiveness of Dox) with increasing risk score, further supporting the model and the use of radiomics in patient selection. The similar proportions of ‘included’ patients in the training and test set (52% and 50% respectively) support the validity of the model and its statistical consistency between the sets [1].

Repeated random draw of 70% of all analyzed patients confirmed the robustness of associations between the final model variables and survival to training/test split, as described in Supplementary Materials.

### 3.6. Model Interpretation

The hazard ratios for the constituent variables in the final model, as shown in Supplementary Table S4, can be used to shed light on the underlying relationships. Here HR > 1 suggests a poor prognostic factor for Dox monotherapy, with its enrichment improving the potential treatment benefit of Evo addition. Most histologies, except for a relatively rare Myxofibrosarcoma, show high HR vs. Leiomyosarcoma, an observation in line with the lack of response to Evosfosfamide in Leiomyosarcoma noted in the original SARC021 trial [5]. Excluding this common histology from the trial does not result in a significant OS benefit in the cohort of the remaining patients, both in the training (*p* = 0.28, HR = 0.80 (0.53–1.20)) or full dataset (*p* = 0.47, HR = 0.88 (0.62–1.24)). A past history of smoking is a trending poor prognostic factor in the model and including only current or ex-smokers in the analysis would result in an improved benefit of Dox + Evo in the training cohort (*p* = 0.12, HR = 0.68 (0.42–1.10)). Interestingly, conversely the never-smokers of the cohort show nearly significantly better survival on standard Dox compared to Dox + Evo (*p* = 0.07, HR = 1.52 (0.96–2.40)). Analysis of the full cohort of trial patients confirmed the relevance of smoking history in the treatment response. For patients without lung metastases, not included in this study, Dox treatment showed no benefit for never-smokers (*p* = 0.89, HR = 0.97 (0.59–1.57)) while a trending benefit of Dox + Evo was observed in Ex/Current smokers (*p* = 0.11, HR = 1.53 (0.92–2.54)). 

**Figure 4:**
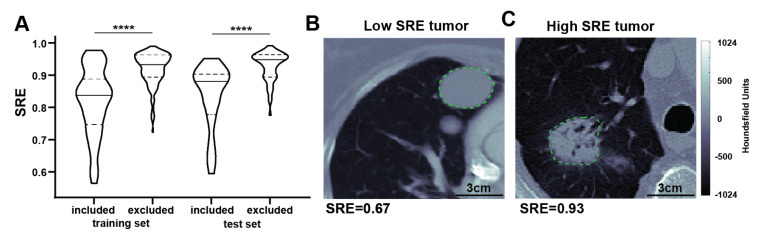
Differences in radiomic features can be apparent visually. The model for selection of patients likely to benefit from Evofosfamide treatment favored low Short Run Emphasis (SRE) radiomic feature for proposed inclusion into the trial. As shown in the violin plot (**A**), significantly lower SRE is observed in the included vs. excluded patient groups both in training and test cohorts. Qualitatively, a representative tumor with low Short Run Emphasis SRE (**B**) appears more regular and homogeneous in a contrast enhanced CT scan than a corresponding tumor with similar volume (15.0 vs. 16.5 mL respectively), and relatively high SRE (**C**), which shows higher intratumor heterogeneity. In the violin plot a solid line indicates median while dotted lines indicate 25th and 75th percentile. **** *p* < 0.0001.

Given the complexity of the question, directly interpreting the imaging information in the model may be challenging. Considering Short Run Emphasis (SRE) individually, analysis shows that the treatment benefit of Dox + Evo is maximized if only patients with tumors of low SRE are included (Supplementary Figure S3). The final multivariable model developed above also favors low SRE values, as shown in Figure 4A for both training and test cohorts. The biological meaning of the SRE feature is not obvious, but inferences can be made. For example, comparing representative tumors with extreme SRE values reveals visual differences. In line with the model, a patient censored after over 2.5 years showed a very low SRE in the lung lesion (Figure 4B) at baseline; and this is visually associated with regularity and homogeneity of the mass. Conversely, another patient deceased on Dox therapy less than 5 months after enrollment presented a lung lesion with high SRE and highly heterogeneous appearance (Figure 4C). While these show extremes, *the value of using a quantitative SRE threshold is to identify those patients whose scans may be less obvious and hence, equivocal*. SRE was not shown to correlate with the CT image characteristics, with Pearson correlation coefficient to in-plane voxel size r = −0.03, and Wilcoxon test *p*-value = 0.70 between the scans of slice thickness ≤3 and >3, supporting the biological origin of the signal. The feature also showed particularly high spatial stability (Concordance coeff. 0.90, 80th percentile).

## 4. Discussion

Herein, we developed a novel radiomic framework, based on the combination of pre-treatment CT data and clinical information, that can provide a treatment-specific model for patient survival prediction in a randomized two-arm trial. This framework was successfully applied to identify STS patients who went on to have a relatively long OS with Dox monotherapy in the SARC021 trial. The strong predictive model of Dox monotherapy response shows significant promise for both clinical care and warrants consideration for prospective validation towards more optimal patient selection in future sarcoma trials with doxorubicin. The sarcoma community has long sought additional efficacious agents in metastatic soft tissue sarcomas with large trials dedicated to alkylators such as ifosfamide [18], and derivatives like palifosfamide [19] that have shown increased response rates but not overall survival benefit. Additionally, albeit in localized soft tissue sarcomas, a recent trial comparing histology-directed therapy compared to doxorubicin concluded that doxorubicin and ifosfamide remained the standard first line agent for all tested histologies [20].

In the SARC021 trial, enrichment for patients unlikely to benefit from doxorubicin would have improved the relative effect of evofosfamide, a compound with renewed clinical interest [21]. These results were successfully validated in the test set and, if applied, the phase 3 trial would have met its primary objective of increased OS with *p* < 0.05. The failure of the SARC021 trial is at least in part due to a shifting OS for Dox monotherapy that is likely multifactorial; inclusive of better patient selection based on histologies, improved supportive care and additionally available subsequent therapies [5,18,19].

Patient selection for drug trials remains a challenge in clinical trial design. In the study, it is notable that the inclusion/exclusion strategy was generated from readily available standard-of-care images and clinical data and can therefore be applied prior to trial start with no protocol changes required. Herein, radiomic methods [16] in combination with novel statistical analysis was used to provide and validate a patient inclusion framework based on widely available standard of care imaging data in a retrospective cohort. While radiomics methods have been used to predict patient survival following different treatments [22,23,24,25], and correlate to tumor hypoxia [26,27], this is the first study to derive the prognostic radiomic features and multivariable models required to discriminate between two arms of an interventional trial. This unique capability to train and implement a strictly treatment-specific model for survival prediction constitutes the main novelty and impact of this work. The proposed general framework can be applied to most solid malignancies to help highlight the drivers of response to particular therapy, especially important in early clinical stages of drug development.

The analysis in this study focused on patients with lung metastases, as they are the most common and deadly metastatic site. This study highlighted the value of combining tumor histology, smoking history, and CT imaging data for trial population enrichment. Notably, neither clinical nor imaging information alone were sufficient to show significant benefit of Evo + Dox in the selected cohort, emphasizing the value of the quantitative model framework proposed in this study, and specific identification of the population of interest. Interestingly, current or ex-smokers were more likely to benefit from the addition of Evofosfamide than those who never smoked. This observation is consistent with the hypoxia-targeting action of the drug, as smoking is known to exacerbate tumor hypoxia through reduction of blood oxygen carrying capacity [28], especially in the lungs, which may lead to improved response to hypoxia targeted treatment in these tumors compared to standard therapy, contributing to the final proposed model. Notably, while there were a number of prognostic features associated with positive outcomes in both groups, there were no features associated with survival in the Dox + Evo cohort independent of the Dox group. This suggests that the biological factors that govern Evo response may not be related sufficiently strongly to the information available from clinical and imaging data. Conversely, the model specific to Dox response as presented in this work, may be prospectively validated and applied directly in upcoming STS trials of doxorubicin treatment. 

A significant strength of the developed model comes from the heterogeneity of the training and testing data. Obtained in a multicenter international trial, the CT imaging was performed on multiple systems with varying acquisition parameters, making the final signature more robust and generalizable than if it were acquired on a single type of instrument or in a single institution. However, there are some limitations to the presented study. First, it is limited to subjects with lung metastases. Although these are common in STS and are the most lethal, it does limit the applicability of this model [7]. While the same radiomic features have been shown to be prognostic in different organ sites, such as Lung and Head&Neck cancers [29], this should be confirmed directly for our model in similar well annotated patient cohort. A mixed advantage of the current approach is a special radiologic protocol was not used for acquisition and, indeed, no planning for this radiomic analysis was considered in the trial design. While this can improve the portability of the model, some level of standardization or qualification would likely have increased the potential statistical power. Going forward, a prospective observational trial could compare the model-calculated risk score to actual OS and to validate the model and understand its biological underpinnings [4]. A prospectively validated model can thus be used to support this radiomic biomarker for patient selection in future trials.

## 5. Conclusions

In summary, in this work we demonstrate for the first time that machine learning can be used to predict differential survival to distinct treatment regimens. We show that radiomic analysis of CT imaging data can be used in combination with clinical information to develop a first of its kind model capable of identifying soft tissue sarcoma patients likely to benefit from novel combination of Doxorubicin + Evofosfamide vs. standard Doxorubicin. Application of the proposed model shows that should patient selection be performed a significant survival benefit could have been observed in an otherwise negative Phase 3 trial. Used prospectively, this approach may in the future improve the chance of determining efficacy of novel therapeutic regimens through better patient selection and guide therapeutic decisions for all metastatic STS through actionable, personalized, image-based, survival prediction.

## Figures and Tables

**Table 1:** Breakdown of patient characteristics. Numbers are presented for each treatment group in training and test cohort. Data are median (IQR) or n (%). *P* value by Wilcoxon test (for age) or Chi squared test (all other variables).

	Training Cohort			Test Cohort		
	Dox + Evo (*n* = 105)	Dox (*n* = 101)	*p*-Value	Dox + Evo (*n* = 47)	Dox (*n* = 43)	*p*-Value
**Age (years)**	60 (47–73)	55 (33–78)	0.06	60 (44–75)	57 (38–76)	0.82
**Sex**			1.00			1.00
Female	59 (56%)	57 (56%)		26 (60%)	24 (51%)	
Male	46 (44%)	44 (44%)		21 (49%)	19 (40%)	
**Smoking history**			0.91			0.46
Never smoker	59 (56%)	55 (54%)		26 (60%)	28 (60%)	
Ever smoker	46 (44%)	46 (46%)		21 (49%)	15 (32%)	
**Primary Tumor Site**			0.89			0.25
Extremity	35 (33%)	40 (40%)		17 (40%)	20 (43%)	
Head/Neck	7 (7%)	5 (5%)		0 (0%)	3 (6%)	
Retroperitoneum	15 (14%)	12 (12%)		8 (19%)	4 (9%)	
Visceral	19 (18%)	17 (17%)		9 (21%)	7 (15%)	
Other	29 (28%)	27 (27%)		13 (30%)	9 (19%)	
**Metastatic sites number**			1.00			0.46
≥2	73 (70%)	71 (70%)		36 (84%)	29 (62%)	
<2	32 (30%)	30 (30%)		11 (26%)	14 (30%)	
**Lung lesions number**			1.00			0.62
>1	82 (78%)	78 (77%)		35 (81%)	29 (62%)	
1	23 (22%)	23 (23%)		12 (28%)	14 (30%)	
**Stage**			0.21			0.46
0	4 (4%)	0 (0%)		1 (2%)	0 (0%)	
Stage I	3 (3%)	6 (6%)		2 (5%)	2 (4%)	
Stage II	24 (23%)	20 (20%)		10 (23%)	16 (34%)	
Stage III	44 (42%)	40 (40%)		16 (37%)	12 (26%)	
Stage IV	30 (29%)	35 (35%)		18 (42%)	13 (28%)	
**Histology**			0.78			0.44
Leiomyosarcoma	44 (42%)	39 (39%)		25 (58%)	17 (36%)	
Epitheloid	1 (1%)	3 (3%)		0 (0%)	0 (0%)	
Liposarcoma	7 (7%)	6 (6%)		0 (0%)	1 (2%)	
Malignant peripheral nerve sheath tumor	4 (4%)	4 (4%)		1 (2%)	4 (9%)	
Myxofibrosarcoma	3 (3%)	4 (4%)		2 (5%)	3 (6%)	
Pleomorphic rhabdomyosarcoma	0 (0%)	2 (2%)		0 (0%)	1 (2%)	
Pleomorphic sarcoma/Malignant fibrous histicytoma	17 (16%)	13 (13%)		9 (21%)	7 (15%)	
Other	29 (28%)	30 (30%)		0 (0%)	1 (2%)	
**Histology Grade**			0.83			0.08
Intermediate	29 (28%)	28 (28%)		21 (49%)	13 (28%)	
Intermediate/High	1 (1%)	2 (2%)		0 (0%)	4 (9%)	
High	75 (71%)	71 (70%)		26 (60%)	25 (53%)	
Unknown	0 (0%)	0 (0%)		0 (0%)	1 (2%)	
**ECOG score**			0.51			0.90
0	58 (55%)	59 (58%)		29 (67%)	25 (53%)	
1	47 (45%)	41 (41%)		18 (42%)	18 (38%)	
2	0 (0%)	1 (1%)		0 (0%)	0 (0%)	
**Prior radiotherapy**			0.55			0.06
No	56 (53%)	59 (58%)		32 (74%)	20 (43%)	
Yes	49 (47%)	42 (42%)		15 (35%)	23 (49%)	
**Prior systemic therapy**			0.41			0.76
No	98 (93%)	90 (89%)		43 (100%)	41 (87%)	
Yes	7 (7%)	11 (11%)		4 (9%)	2 (4%)	

## Data Availability

Analysis code and preprocessed imaging and clinical data will be made available on https://github.com/mrtomasz91/SarcEnrichment repository (accessed on 20 December 2021).

## Supplementary Materials

The following are available online at https://www.mdpi.com/article/10.3390/tomography8010028/s1, Figure S1: Feature robustness to tumor segmentation. For all tumors in the training cohort, radiomic features were calculated for the original ROI as segmented by the radiologist, as well as the same ROI shrunk or dilated radially by 1 mm. Concordance Coefficient for feature values between the ROIs is presented in the heatmap, with each line representing a radiomic feature, showing differences between feature types, as indicated on the left. GLCM—Grey Level Co-occurrence Matrix, GLRLM—Grey Level Run Length Matrix, GLSZM—Grey Level Size Zone Matrix, NGTDM—Neighboring Gray Tone Difference Matrix; Figure S2: Clinical model performance. Including only the tumor histology and patient smoking history information in a multivariate Cox model, a risk score threshold was identified for a significantly (*p* < 0.05) different survival in the two treatment groups (A). The graph of corresponding treatment Hazard Ratios (HR) for the considered threshold values (B) shows an increasing benefit of Evo with exclusion of low-risk score patients, and 48% of the original training cohort included at the optimal threshold. Solid red line indicates p = 0.05 significance level and dashed red line indicates the optimal threshold value and corresponding p and HR; Figure S3: Single feature model performance. Considering the value of Short Run Emphasis (SRE) radiomic feature as a threshold for inclusion, the graph of p value of survival differences between treatment arms depending on the threshold value is presented in (A), while the corresponding Hazard Ratio plotted against a percentage of patients included at this threshold is shown in (B). The graphs on the left describe the approach where patients with SRE below the threshold are included in the analysis, showing an improved survival of Evo treated patients (HR < 1), while the graphs on the right, patients with SRE over the threshold are considered, favoring Dox treatment (HR > 1). Solid red line indicates p = 0.05 significance level and dashed red line indicates the optimal threshold value and corresponding p and HR; Figure S4: Tumor volume cannot be used for patient selection. Graphs of p value of survival differences between treatment arms in the treatment cohort depending on the volume threshold value are presented for patient cohort below the maximum volume threshold (A) and above minimum volume threshold (B). Neither of these graphs show points reaching 0.05 value, as indicated by red line. The corresponding Hazard Ratio values for Dox + Evo treatment depending on the percentage of entire training cohort are shown in (C) and (D) for patient inclusion below and above threshold value respectively; Table S1: Description of clinical features; Table S2: Correlation to volume and spatial stability of radiomic features. The Pearson correlation coefficient and concordance coefficient for spatial stability analysis are shown for all radiomic features; Table S3: Association with survival in radiomic features and clinical covariates by treatment arm. Univariable Cox regression model was applied separately in the Doxorubicin + Evofosfamide (Dox + Evo) and Doxorubicin only (Dox) treatment arms to calculate the p value (‘p Evo’ and ‘p Dox’ respectively) and hazard ratio (‘HR Evo’ and ‘HR Dox’ together with 95% Confidence Intervals) for the relationship of each feature and covariate with overall survival. P values below 0.05 are highlighted in red, while these above 0.30 are highlighted in green. Hazard Ratios for categorical variables were calculated against most common category; Table S4: Multivariable Cox model. The Hazard Ratios (HR) together with 95% Confidence Intervals (CI) and the associated p values (log-rank test) in the multivariable Cox regression model, in the Doxorubicin arm of the training cohort. The model was further applied to identify patients expected to benefit from Doxorubicin monotherapy. Hazard Ratios for categorical variables were calculated against most common category.
